# Molecular characterization of *Trichinella spiralis* galectin and its participation in larval invasion of host’s intestinal epithelial cells

**DOI:** 10.1186/s13567-018-0573-3

**Published:** 2018-08-02

**Authors:** Jia Xu, Fan Yang, Da Qi Yang, Peng Jiang, Ruo Dan Liu, Xi Zhang, Jing Cui, Zhong Quan Wang

**Affiliations:** 1 Department of Parasitology, Medical College, Zhengzhou University, Zhengzhou, 450052 China; 2 School of Life Science, Zhengzhou University, Zhengzhou, 450052 China

## Abstract

The aim of this study was to study the molecular characteristics of *Trichinella spiralis* galectin (Tsgal) and interactions between Tsgal and host’s intestinal epithelial cells (IECs). The functional domain of Tsgal was cloned and expressed in an *E. coli* system. The Tsgal was 97.1% identity to the galectin of *T. nativa* and 20.8% identity to the galectin-8 of humans. Conserved domain analysis revealed that Tsgal belongs to TR-type galectin and has two carbon recognized domain. The rTsgal with 29.1 kDa could be recognized by *T. spiralis*-infected mice at 42 days post-infection (dpi). The transcription and expression of Tsgal gene was detected by RT-PCR and Western blotting in all *T. spiralis* developmental stages (intestinal infective larvae, adult worms, newborn larvae, and muscle larvae). The IFA results revealed that Tsgal was mainly located at the cuticles and stichosomes of *T. spiralis* larvae (ML, IIL and NBL). The rTsgal had hemagglutinating function for erythrocytes from human, rabbit and mouse. The results of Far Western blot and confocal microscopy indicated there was specific binding between rTsgal and IECs, and the binding was located the membrane and cytoplasm of the IECs. Out of four sugars (sucrose, glucose, lactose and maltose), only lactose was able to inhibit the rTsgal agglutinating role for human type B erythrocytes. Moreover, the rTsgal could promote the larval invasion of IECs, while the anti-rTsgal serum inhibited the larval invasion. These results demonstrated that Tsgal might participate in the *T. spiralis* invasion of intestinal epithelium in early infection stage.

## Introduction

Trichinellosis is an important zoonotic disease resulted from the parasitic nematode *Trichinella* [1]. This zoonosis is widely distributed in many kinds of mammalian species, including humans [2]. Human infections are mainly due to eating raw or uncooked animal meat infected with *Trichinella spiralis* [3–5]. Since outbreaks of human trichinellosis has been reported in many countries, this disease has become a public health concern and regarded as a re-emerging or emerging disease [6, 7]. Thus, to prevent the swine from *Trichinella* infection is necessary for ensuring meat food safety and the public health [8, 9].

*T. spiralis* muscle larvae (ML) are liberated from the capsules in stomach after infected meat is digested by gastric fluid, and then develop into intestine infective larvae (IIL) after activation by bile and enteral contents at 0.9 hours post-infection (hpi) [10, 11]. The IIL larvae penetrate into the intestinal epithelium, and undergo the four molts to develop to adult worms (AW) at 31 hpi. After mating in the intestine, the female adults shed newborn larvae (NBL) which enter the venules and lymphatic vessels, and then spread throughout the body via blood circulation until they reach the final parasitizing skeletal muscles and develop into the encapsulated ML [12]. The ML can survive from about 1–2 to 10–15 years in host without obvious damage [13, 14]. However, the mechanism of *T. spiralis* invasion of intestinal epithelium has not been clarified completely. The studies on larval invasion mechanism will be valuable to develop the novel vaccine candidate and drug targets against *Trichinella* infection [8].

Galectins are characterized by a unique carbon recognized domain (CRD) sequence motif binding to β-galactoside, and constitute an evolutionary conserved family. They are ubiquitous and structurally in eukaryotic taxa, including vertebrate, invertebrate, fungus and sponges [15]. Based on structural features, three types of galectin proteins have been identified, including proto-type, chimera-type and tandem repeat–type (TR) [16]. Proto-type galectins are homodimers containing a single CRD without covalent bond. The chimera types have a C-terminal CRD with the opposite N-terminal domain rich in proline and glycine. While the TR-type consisted of two CRDs which are connected with a functional linker peptide. The subtypes of mammalian galectins were named according to the order of their discovery. Thus far, 15 galectins have been identified in mammal, including nine proto-type (galectins-1, -2, -5, -7, -10, -11, -13, -14 and -15), five TR-types (galectins-4, -6, -8, -9, -12) and only one chimera-type (galectin-3) [17].

Several galectins or galectin-like proteins have been described in some parasites. They are speculated to play a role in the invasion process and immunomodulation. The galectin derived from *Dirofilaria immitis* had been demonstrated to active the host fibrinolytic system and stimulate the proliferation of smooth muscle cells and degradation of the extracellular matrix (ECM), which is related to the parasite surviving in the host [18]. In the experimental autoimmune encephalomyelitis (EAE) model, treatment with galectin from *Toxascaris leonine* could enhance the EAE severity and antibody production which resulted in the attenuation of EAE remission [19]. However, another previous study on inflammatory bowel disease (IBD) model demonstrated that galectin of *Toxascaris leonine* deliver a beneficial effect on dextran sodium sulfate (DSS) by exhibiting significantly increase of the levels of TGF-β and IL-10 [20]. The expression level of galectin-10 mRNA of *Angiostrongylus cantonensis* was upregulated after being stimulated with H_2_O_2_, which indicated the natural galectin can be induced under the reactive oxygen stress [21]. The recombinant galectins from *Haemonchus contortus* had been indicated to be a potential vaccine target to protect goats from *H. contortus* infection [22]. However, studies on biological characteristics and functions of *T. spiralis* galectins have not been reported in the references available up to date.

One galectin from *T. spiralis* (Tsgal, GenBank accession No. EFV62290) has been identified in the ML surface protein by immunoproteomics [23, 24]. The aim of this study is to identify the biological characteristics and functions of Tsgal in *T. spiralis* larval invasion of host’s intestinal epithelial cells.

## Materials and methods

### Bioinformatics analysis of Tsgal

The bioanalysis software and websites are used to analyze the sequence of Tsgal as described [25, 26]. The physical and chemical properties of amino acid sequence of Tsgal were predicted through Protparam Server, the signal peptide was predicted by using SignalP 4.1 Server, and the transmembrane domain was predicted on TMHMM Server v. 2.0. The sequence was submitted to BcePred Prediction Server to predict the antigenic epitopes of Tsgal. The domain analysis of Tsgal was performed using Conserved Domain database of NCBI. The amino acid sequence of Tsgal was alignmented with galectins from other organisms using the clustal X. Galectin sequences from other species were downloaded from NCBI. The GenBank accession numbers of other galectins are as follow: *T. nativa* (KRZ49476), *T. murrelli* (KRX35770.1), *T. nelsoni* (KRX15368.1), *T. patagoniensis* (KRY16872.1), *T. pseudospiralis* (KRY89534.1), *Trichinella* sp. T6 (KRX72992.1), *Trichinella* sp. T8 (KRZ90044.1), *Trichinella* sp. T9 (KRX52357.1), *T. zimbabwensis* gal lec-3 (KRZ13804.1), *Trichostrongylus colubriformis* (AAD11971.1), *Caenorhabditis elegans* (AAB87718.1), *Caenorhabditis elegans* (NP_496159.1), *Brugia malayi* (AAF37721.1), *Haemonchus contortus* (AAB88823.1), *Onchocerca volvulus* (AAD00843.1), *Dirofilaria immitis* (AAF37720.1), *Toxascaris Leonina* (4HL0_B Chain B, Crystal Structure Of Full-length), *Homo* gal-8 (AAF19370) and *Mus* gal-6 (NP_034837). The phylogenetic tree of these galectin sequences was constructed using MEGA7.0. The phylogeny was constructed by using the maximum parsimony method [27].

### Parasite, experimental animal and cells

*Trichinella spiralis* strain (ISS534) was isolated from domestic pigs in central China, this strain was maintained in our department by passage in mice. Female BALB/c mice aged 35 days old were purchased from the Henan Provincial Experimental Animal Center. The intestinal epithelial cells (IEC) were isolated from the intestines of normal BALB/c mice in our laboratory [28] and the primary culture cells at passage 8 were used for the invasion assay. Previous studies have showed that the IECs are susceptible to *T. spiralis* invasion [29]; whereas the mouse striated muscle myoblast cells (C2C12) were resistant to the invasion and applied as negative control [29].

### Worm collection and protein preparation

The ML were recovered at 42 days post-infection (dpi) by artificial digestion of infected mouse carcasses with 0.33% pepsin and 1% HCl as described [30]. *T. spiralis* IIL were collected from mouse small intestines at 6 hpi [24], and intestinal AW were collected at 3 and 6 dpi as previously described [31]. The NBLwere collected from the 6 dpi adult females which was cultured in 5% CO_2_ at 37 °C for 18 h in the PRMI-1640 medium containing 10% fetal bovine serum (FBS; Gibco) [32]. The excretory/secretory (ES) proteins of ML and soluble crude somatic proteins of ML, IIL, AW, and NBL were prepared [33, 34]. The protein concentration of ES proteins and somatic proteins was measured by the Bradford method [35].

### Cloning, expression of recombinant Tsgal protein

Total RNA was obtained from the ML with Trizol (Invitrogen, USA) according the standard operation instructions supplied by the manufacure. The first-strand synthesis of cDNA was performed using AMV reverse transcriptase (Promega, USA). Tsgal gene was amplified using PCR with specific primers carrying BamHI and HindIII restriction enzyme sites (in italics) (5′-CACCATCACCATCAC*GGATCC*AAAGTTCCGTATTTAGCCAA-3′, 5′-CAAGCTCAGCTAATT*AAGCTT*TCATTCTAAATGAATCAACT-3′). PCR products were cloned into the prokaryotic expression vector pQE-80L vector using ClonExpress™ II one-step clon kit (Vazyme, China) following the instruction, the recombinant plasmid pQE-80L/Tsgal was then transferred into the *Escherichia coli* BL21 (DE3) (Novagen). The expression of rTsgal protein was induced for 6 h at 37 °C by using 0.5 mM IPTG. The rTsgal was found in the supernatant, subsequently purified with Ni-NTA-Sefinose resin (Sangon Biotech Co., Shanghai, China) [36]. The concentration of purified rTsgal was determined, then analyzed by SDS-PAGE with 12% acrylamide separating gel [37].

### Preparation of anti-rTsgal serum

Fifty BALB/c mice were immunized by subcutaneous injection of 20 μg rTsgal protein emulsified with complete Freund’s adjuvant. Three boost immunizations were performed with the same amount of rTsgal emulsified with incomplete Freund’s adjuvant at 10-day intervals [14, 38]. The immunized mice were bled on the 7th day after the last immunization, and the sera were isolated.

### Western blot analysis

Samples containing somatic proteins of ML, IIL, AW and NBL, ML ES proteins and rTsgal protein were separated by SDS-PAGE with a 12% separating gel and then were transferred onto the polyvinylidene difluoride (PVDF) membranes using a Mini Trans-Blot^®^ Cell (Bio-Rad, China) at 250 mA for 1.5 h [39]. The membrane was sliced into strips and then blocked for 2 h at room temperature by 5% skim milk in tris-buffered saline—0.05% Tween-20 (TBST). After washing with TBST for three times, the strips were incubated at 37 °C for 1 h with 1:100 dilutions of different sera (anti-rTsgal serum, *T. spiralis*-infected mouse serum and pre-immune serum). Following washing, strips were incubated at 37 °C for 1 h with 1:5000 dilutions of anti-mouse IgG-HRP-conjugate (Southern Biotechnology, USA). After the final washes, the strips were colored with 3, 3′-diaminobenzidine tetrahydrochloride (DAB; Sigma) [40, 41].

### RT-PCR analysis of Tsgal gene transcription

Total RNA was extracted from *T. spiralis* different phases, including IIL, AW, NBL and ML. The transcription of Tsgal gene at each stage was observed by RT-PCR as described [29]. The positive control was performed with amplifying *T. spiralis* GAPDH (GenBank accession No. AF452239).

### Immunofluorescent assay (IFA)

IFA was used to locate the position of Tsgal protein in *T. spiralis*. The whole ML, IIL, AW and NBL were fixed with acetone, the ML and AW were also fixed in paraformaldehyde and embedded in paraffin. Then 2-μm sections of this nematode were prepared by using the microtome-cutting. The whole parasites and sections were first blocked with 5% goat serum in PBS and then incubated for 1 h at 37 °C with anti-rTsgal serum diluted at 1:10 [42]. Pre-immune normal serum was utilized as negative control and infection serum as positive control. Following washes, the whole parasites and sections were incubated with 1:100 dilutions of a goat anti-mouse IgG-FITC conjugate (Santa Cruz, USA). Finally, the parasites and sections were washed for three times with PBS, and observed with a fluorescent microscope (Olympus, Japan) [43].

### Far Western analysis

In order to analyze the protein interaction between Tsgal and IECs, the protein samples of IECs were prepared and separated by SDS-PAGE with 12% resolving gels as described [44]. After electrophoresis, the IEC proteins were transferred to PVDF membrane, and incubated with 20 μg/mL of the rTsgal for 1 h at 37 °C. The negative control was performed by using PBS instead of rTsgal protein. The membrane was first probed by the primary antibodies (anti-rTsgal serum, mouse infection serum or pre-immune serum) at 37 °C for 1 h. After washing with PBS for three times, the membrane was then probed with the anti-mouse IgG-HRP conjugate as secondary antibodies at 37 °C for 1 h, and lastly dyed with the 3,3′-diaminobenzidine tetrahydrochloride (DAB, Sigma) [45].

### Cell immunostaining and confocal microscopy

For confocal microscopy, the IECs were cultured in a 6-well cell culture plate. After cells were grown to confluence, the IECs were fixed in acetone for 10 min and blocked for 2 h at 37 °C with 5% goat serum for 30 min, subsequently incubated with 20 μg/mL rTsgal (PBS as negative control). After being rinsed, the IECs were probed at 37 °C for 1 h by using anti-rTsgal serum, infection serum or pre-immune serum. After washes again, the IECs were probed by anti-mouse IgG-FITC conjugate at 37 °C for 1 h [44]. Imagines were captured by using an Olympus FV1200 laser scanning microscope and analyzed by using Olympus Fluoview software.

### Hemagglutination activity assay and sugar inhibition assay

The hemagglutination activity of galectins may play a key role for affecting cellular activation and function through sugar binding- dependent mechanisms in the process of parasite invasion of hosts [20, 48]. To determine the Tsgal activity, hemagglutination and sugar-binding assay were used in this study. Human blood samples with A, B, O and AB types were generously supplied by the Henan Red Cross; blood samples were also collected from rabbit  and mice in this study. Erythrocytes were centrifuged at 350*g* for 10 min, followed by washing with pH 7.0 sterilized saline solution for three times. Then, the erythrocytes were re-suspended in 2% saline solution. Hemagglutination activity assay were conducted as described with slightly modification [46, 47]. First, 25 μL of different dilutions (0–400 μg/mL) of the rTsgal was added into the 96-well plate, and then 25 μL of 2% suspensions of erythrocytes were also added to each well. The experiment was carried out in triplicate. The plate was incubated in the room temperature for 1 h, complete agglutination was observed by naked eye, the lowest dose of the rTsgal for inducing agglutination was recorded.

Sugar inhibition assay was performed according to the previous studies [48] with slight modification. Four different carbohydrates including lactose, glucose, maltose and sucrose were used in our study. The rTsgal (100 μg/mL) was added to the 96-well plates, 25 μL different dilutions (100–400 mM) of carbohydrates were subsequently added into the well. Following being incubated for 1 h at the room temperature, 2% suspensions of type B human erythrocytes were added and incubated in the room temperature another 1 h to observe the hemagglutination. Two control experiments were conducted without carbohydrate or rTsgal which was replaced with PBS, respectively.

### The in vitro invasion assay

The in vitro *T. spiralis* IIL invasion assay was performed to evaluate the effects of anti-rTsgal serum on larval invasion of intestinal epithelium [49, 50]. In brief, The ML was activated into the IIL by 5% swine bile at 37 °C for 2 h [11]. The IEC cell monolayers were overlaid with 100 IIL suspended in the semisolid medium (DMEM supplemented with l-glutamine, 15 mM HEPES, 1.75% agarose). The media were supplemented with different dilutions (1:10–1:800) of anti-rTsgal serum, pre-immune serum or infection serum. After incubation at 5% CO_2_ at 37 °C for 2 h, the larval invasion of monolayer was examined and counted. The larvae still suspended in the semisolid medium was counted as non-invaded larvae, while the invaded and migrated in the monolayer were defined as invaded larvae [51]. Besides, to investigate the effects of the rTsgal protein on the larval invasion, different concentration (0–15 μg/mL) of the rTsgal was also used in the invasion assay [29].

### Statistical analysis

The data were statistically analyzed with the aid of SPSS 17.0 soft-ware. The values were presented as mean ± standard deviation (SD). The differences of the percentage of invaded larvae in IECs among three groups of different serum were compared by using Chi square test. The association between larval invasion and rTsgal dose was analyzed by a linear regression. Differences of larval invasion rate in the presence or absence of rTsgal were compared using one-way ANOVA. The level of statistical difference was *P* < 0.05.

## Results

### Bioinformatics analysis of Tsgal

The complete CDS of Tsgal (XP_003381656.1) contains 855 bp encoding 284 amino acids. Signal P 4.1 Server prediction showed that there is a signal peptide at the first 15^th^ amino acids. TMHMM server analysis revealed no transmembrane domain of Tsgal. The Tsgal had seven potential antigenic epitopes (aa 85–95, 108–112, 153–160, 167–170, 201–209, 220–225 and 230–234). Conserved domain analysis revealed that the Tsgal had two CRD located at 23–150 aa and 165–282 aa, and identified as TR-type. In order to amplify the complete functional domain of Tsgal gene, a pair of specific PCR primers were designed to amplify the fragment with 61–855 bp in this study, and this fragment was 795 bp and encoded 264 amino acids with a molecular weight (MW) of 29.1 kDa.

### Sequence alignment and phylogenetic analysis of Tsgal

**Figure 1:**
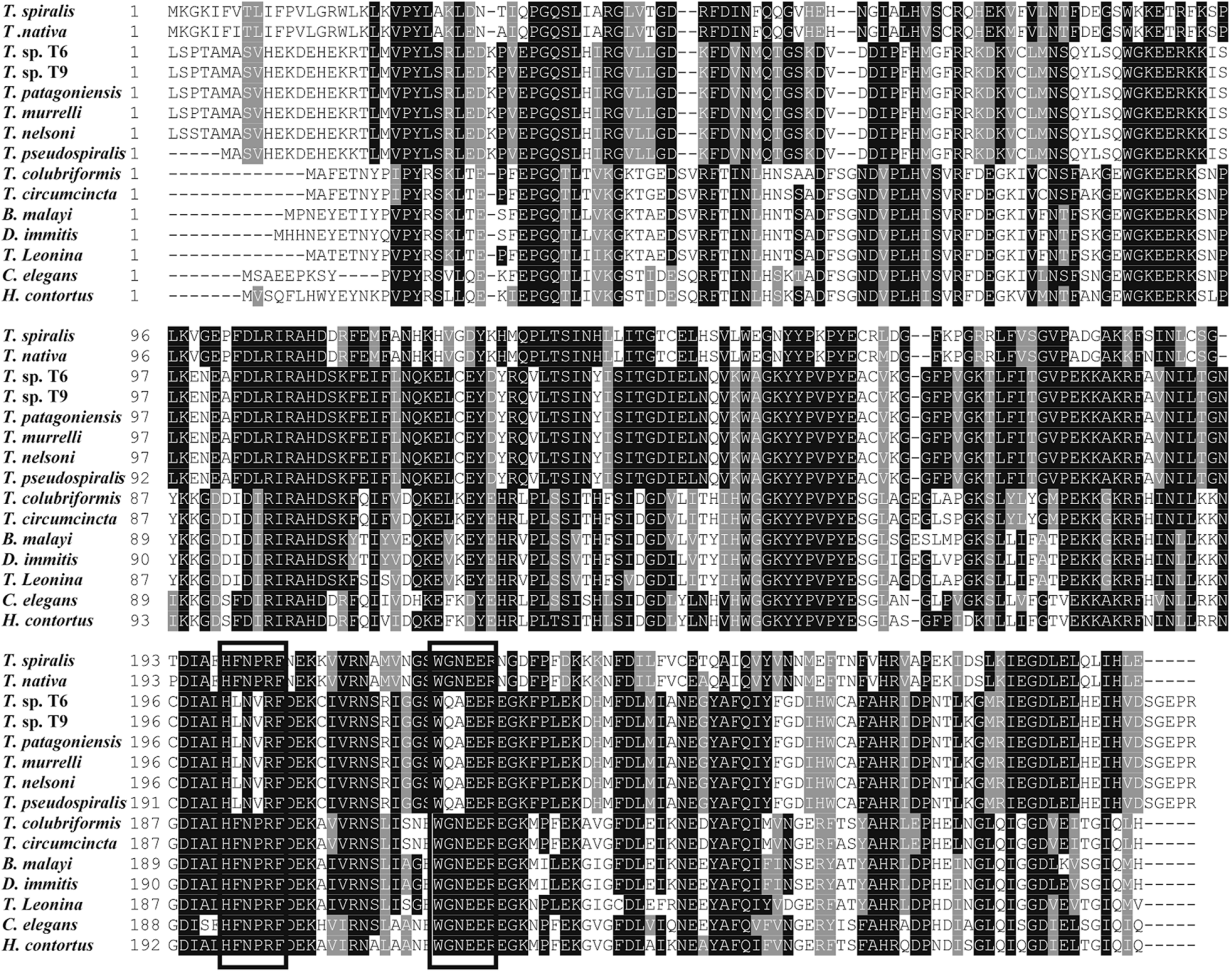
**Sequence alignment of** ***Trichinella spiralis*** **galectins (XP003381656.1) with other** ***Trichinella*** **species and other organisms.** The multiple sequence alignment was performed in the Clustal X and displayed using BOXSHADE. The conserved regions HFVRF and WGXEXR motifs of galectins were shown in box. Black shade indicated that residues identical to Tsgal, and conservative substitutions were shaded grey.

**Figure 2:**
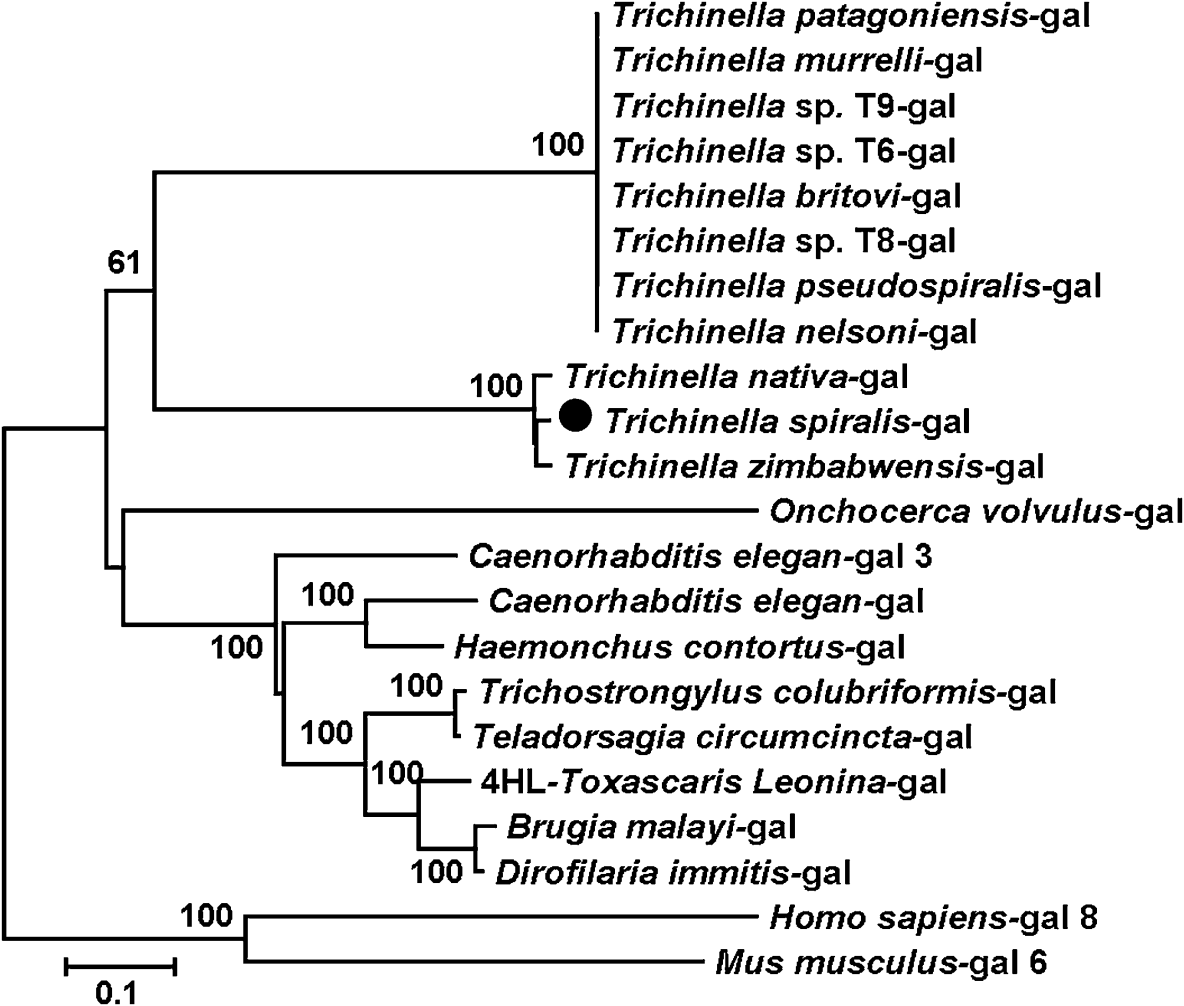
**Phylogenetic tree of analysis of Tsgal.** Phylogenetic relationship of Tsgal with galectins of other nematodes, mouse and human with maximum parsimony method and drawn with MEGA. Bootstrap values that are higher than 60 are indicated on branches. The sequence indicated with solid circle was Tsgal protein (XP003381656) cloned in this study.

The homology comparison of Tsgal amino acid sequences with those of several nematodes, mouse and human was shown in Figure 1. The conserved motifs HFNPRF and WGXEXR were located in the C-terminal CRD. The amino acid sequences of the Tsgal from *Trichinella spiralis* had 97.1% identity with the galectin of *T. nativa*, 68.8% identity with gal lec-3 of *T. zimbabwensis.* At different genus level, Tsgal was found to be 41.4% identity with galectin of *Haemonchus contortus*, and 36.0% identity with the galectin of *Brugia malayi*. Furthermore, some galectin subtypes of mouse and human were also compared with Tsgal, 7.8–22.1% similarity was found between Tsgal and galectin subtypes of human and mice (Table 1). The phylogenetic analysis of the galectins of various organisms supported the monophyletic group of three species of *Trichinella* (*T. spiralis*, *T. nativa* and *T. zimbabwensis*) (Figure 2).

**Table 1:** Similarity (%) of amino acid sequences of Tsgal to various galectins of human and mouse

Hosts	gal-1	gal-2	gal-3	gal-4	gal-6	gal-7	gal-8	gal-9	gal-10	gal-12	gal-13	gal-16
Human	12.3	11.4	15.9	19.7	–	12.7	20.8	17.9	7.8	14.8	8.9	9.9
Mouse	12.3	11.4	15.6	18.9	22.1	12.7	19.1	17.9	–	14.8	–	–

– Represent no sequence was recorded in NCBI.

### Expression and purification of rTsgal protein

**Figure 3:**
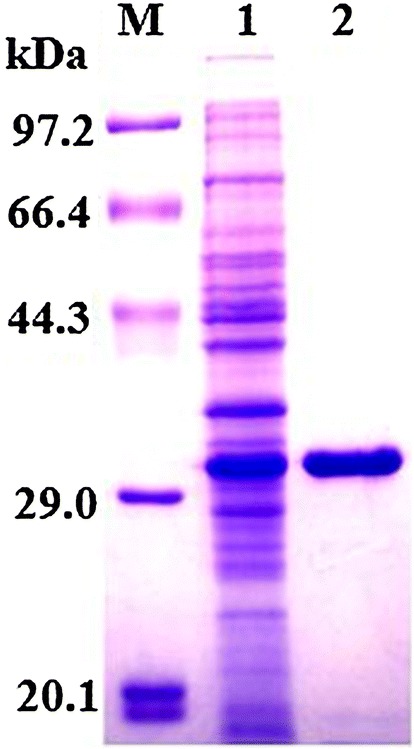
**SDS-PAGE analysis of rTsgal.** Lane M: the protein molecular weight marker; lane 1: lysate of BL21 bacteria harboring pQE-80L/Tsgal after induction; lane 2: the purified recombinant Tsgal.

A 795 bp amplified fragment was obtained and cloned into the pQE-80L expression vector using seamless clone kit. After induction with IPTG, the fusion protein with the His-tag was expressed in *E. coli* BL21 harboring pQE-80L/Tsgal. The rTsgal protein was purified by using Ni-NTA-Sefinose Column and a single band was observed on SDS-PAGE analysis. The MW of the rTsgal is approximately 29.1 kDa which was congruent with its predicted size (Figure 3).

### Identification of rTsgal by Western blot analysis

**Figure 4:**
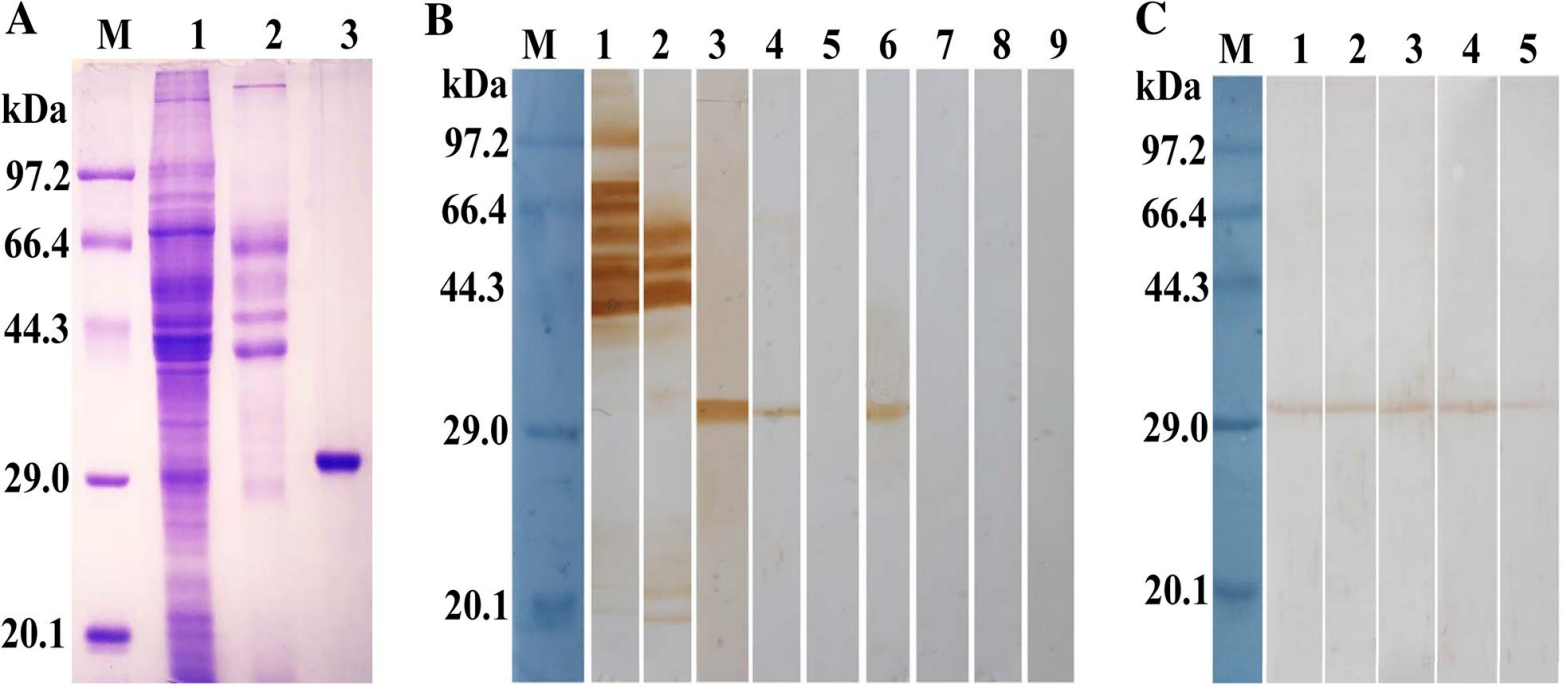
**Identification of recombinant Tsgal protein.** **A** SDS-PAGE analysis of the rTsgal. Lane M: The protein molecular weight marker; lane 1: *T. spiralis* ML somatic proteins; lane 2: ML ES protein; lane 3: the purified rTsgal. **B** Western blot analysis of the rTsgal antigenicity. ML somatic proteins (lane 1), ML ES proteins (lane 2) and the rTsgal (lane 3) were probed with mouse infection sera. By using anti-Tsgal serum, the natural Tsgal protein was found in ML somatic proteins (lane 4), but not in ML ES proteins (lane 5), and rTsgal (lane 6) was also probed by anti-Tsgal serum. The ML somatic proteins (lane 7), ES proteins (lane 8) and rTsgal (lane 9) were not probed with normal mouse serum. **C** Western blot analysis indicated that native Tsgal was recognized by anti-Tsgal serum in somatic proteins of all lifecycle stages of *T. spiralis* (lane 1: ML; lane 2: IIL; lane 3: 3 days AW; lane 4: 6 days AW, lane: NBL).

Western blot results revealed that the rTsgal protein was probed by anti-rTsgal serum and *T. spiralis*-infected mouse serum. By using anti-rTsgal serum the natural Tsgal protein was identified in somatic proteins of ML, IIL, 3 days AW, 6 days AW and NBL, but not in ML ES protein (Figure 4), demonstrating the Tsgal is one component of somatic proteins of this parasite, but not ES protein of the ML.

### RT-PCR analysis of Tsgal gene transcription

**Figure 5:**
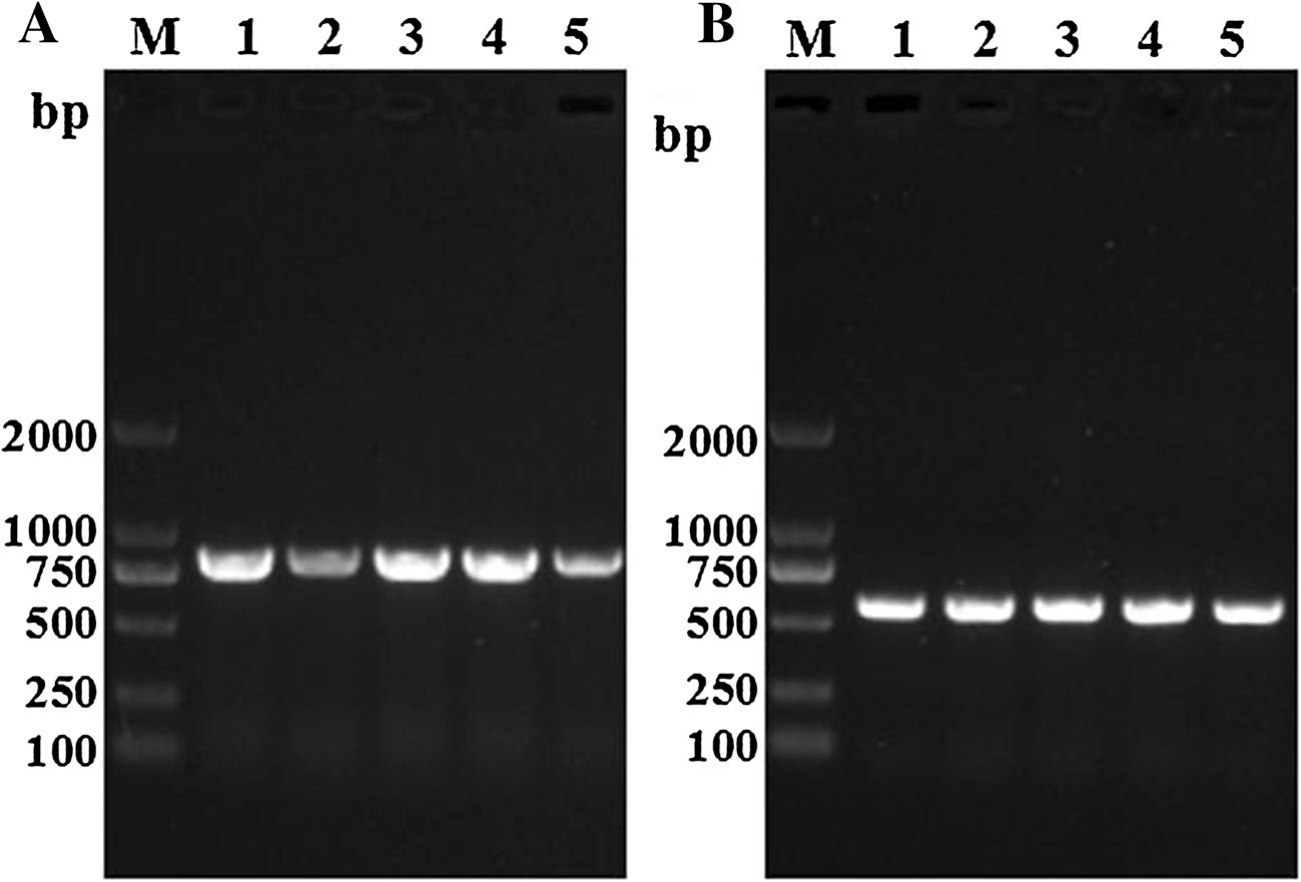
**RT-PCR analysis of Tsgal** **A and GAPDH B transcription at** ***Trichinella spiralis*** **different developmental phases.** lane M: DL2000 DNA marker; lane 1: ML; lane 2: IIL at 6 hpi; lane 3: 3 dpi AW; lane 4: 6 dpi AW; lane 5: NBL.

The Tsgal mRNA transcript (980 bp) was observed at all *T. spiralis* lifecycle phases (e.g., ML, 6 h IIL, 3 and 6 days AW, and NBL). The expected band (570 bp) of GAPDH was also generated in all of the samples (Figure 5).

### Expression and immunolocalization of Tsgal at various phases

**Figure 6:**
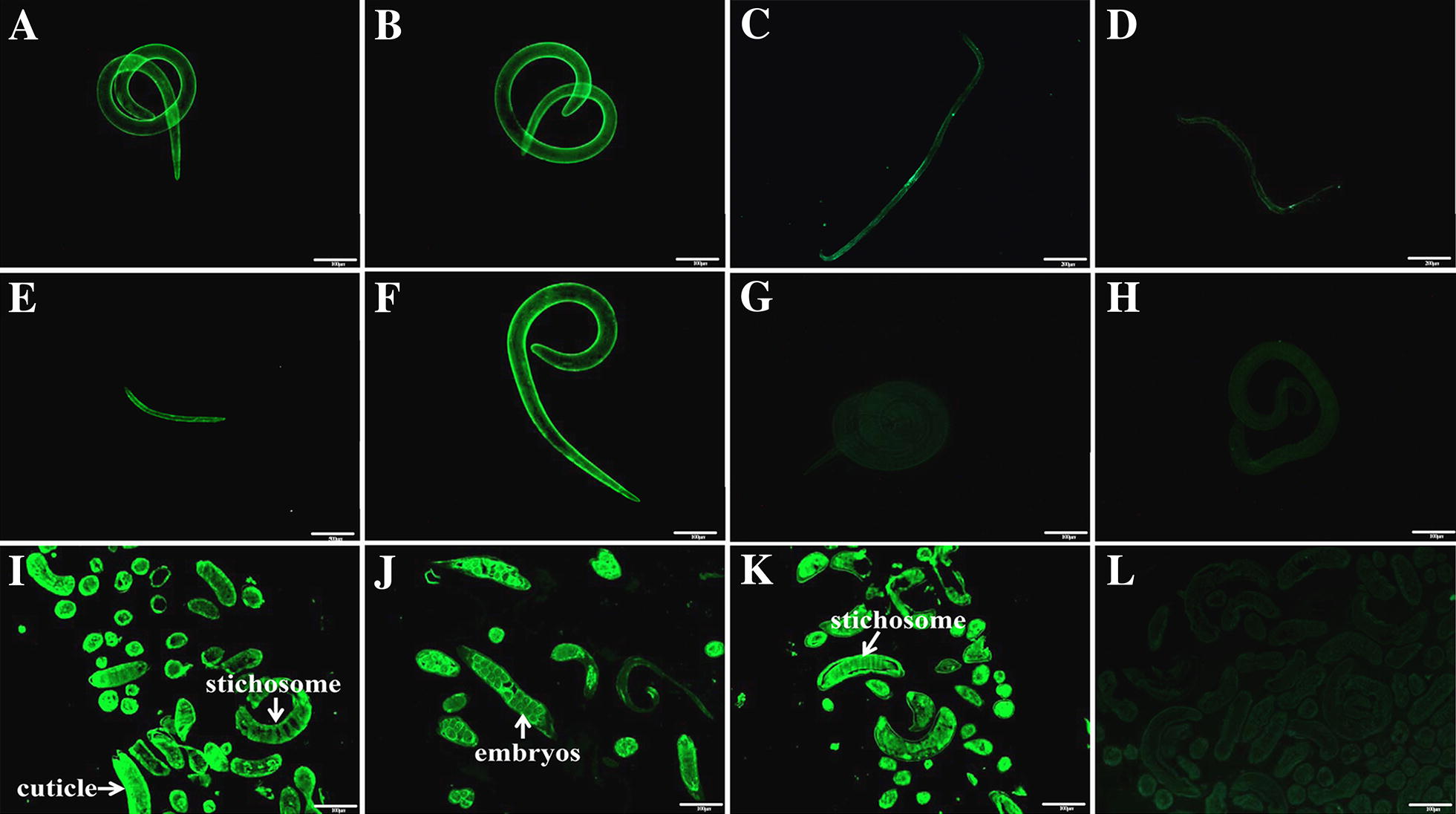
**Expression and immunolocalization of Tsgal at** ***T. spiralis*** **various developmental phases.** **A-E**: The results of IFA with intact nematodes probed by anti-rTsgal serum. The intense immunostaining was observed on the surface of ML **A**, IIL **B** and NBL **E**, but slight staining was found on the surface of 3 days male (**C**) and female (**D**). The ML (**F**) recognized by infection serum was served as a positive control; the ML incubated with normal serum (**G**) and PBS (**H**) was served as negative control. **I**–**L** Immunolocalization of Tsgal in *Trichinella spiralis* ML and AW using IFA with worm sections. By using anti-Tsgal serum the intense immunostaining was located at the cuticles and stichosomes of the ML (**I**), and embryos of female adult at 3 dpi (**J**). The section of ML (**K**) recognized by infection serum as a positive control; The ML (**L**) not recognized by normal serum as negative controls. Scale-bars: **A**, **B**, **F**–**L**, 200 μm; **C**, **D** 100 μm; **E**, 500 μm.

The results of IFA with the whole nematode revealed that by using anti-Tsgal serum the intense immunofluorescent staining was found on the surface of the ML, IIL and NBL, weak staining was observed on the surface of AW (Figure 6). While the nematode sections were probed with the anti-rTsgal serum, intense staining was detected at the cuticles and stichosomes of the ML. The immunostaining was also observed at the cuticle and embryos of female adult at 3 dpi.

### Far Western analysis of binding of rTsgal with IEC proteins

**Figure 7:**
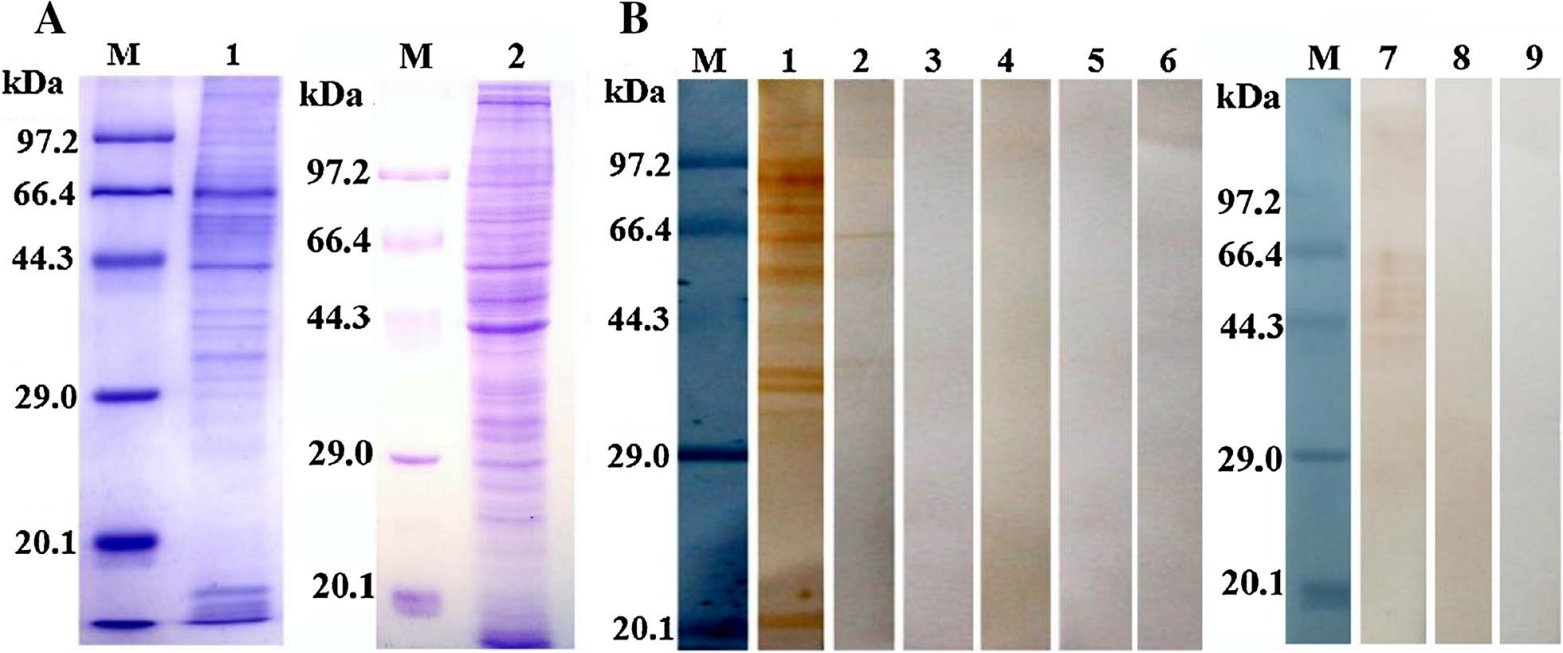
**Far Western blot analysis of rTsgal binding to IEC proteins.** **A** SDS-PAGE analysis of the IEC proteins. Lane M: The protein molecular weight marker; lane 1: The IEC proteins; lane 2: The C2C12 lysates. **B** Far Western analysis of the rTsgal binding to IEC proteins. The IEC proteins blotted on the PVDF membrane was pre-incubated with rTsgal (lane 1–3), PBS (lane 4–6), C2C12 proteins was also pre-incubated with rTsgal (lane 7–9). Subsequently, both cell proteins were incubated with anti-rTsgal serum (lane 1, 4 and 7), infection serum (lane 2, 5 and 8) or normal serum (lane 3, 6 and 9), respectively. The binding between rTsgal and IECs was detected only with anti-rTsgal serum (lane 1) and infection serum (lane 2).

The IEC lysates were firstly analyzed with SDS-PAGE, and the results showed normal IEC proteins have about 22 bands with a molecular weight of 15.2–97.2 kDa (Figure 7A). Far Western results showed that ten bands (20.5–97.2 kDa) of IEC lysates pre-incubated with rTsgal were probed by anti-rTsgal serum, and the majority of recognized bands have a MW of more than 30 kDa (Figure 7B). However, only one band with 65.3 kDa of IEC lysates was recognized by mouse infection serum. No bands of C2C12 lysates pre-incubated with rTsgal were probed by either anti-Tsgal serum or infection serum.

### IFA and confocal microscopy of binding and cellular localization of rTsgal with IECs

**Figure 8:**
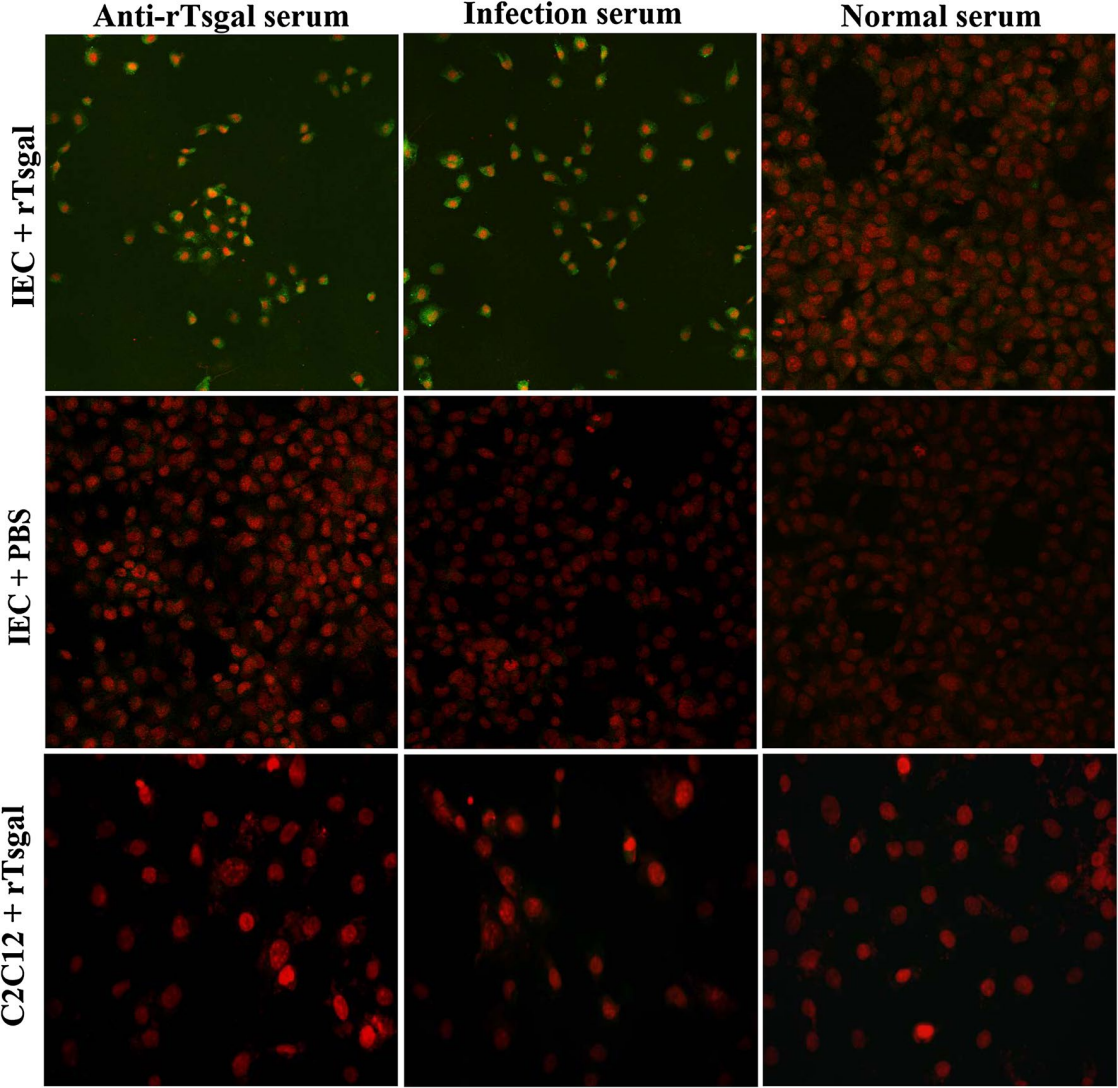
**Immunofluorescent assay of rTsgal binding to IECs (× 200).** The IECs were pre-incubated with rTsgal or PBS, the C2C12 s were pre-incubated with rTsgal at 37 °C for 2 h. Then, IECs were probed by anti-rTsgal serum, infection serum or normal serum, finally stained by using anti-mouse IgG-FITC conjugate, and the cell nuclei were dyed red with propidium iodide.

**Figure 9:**
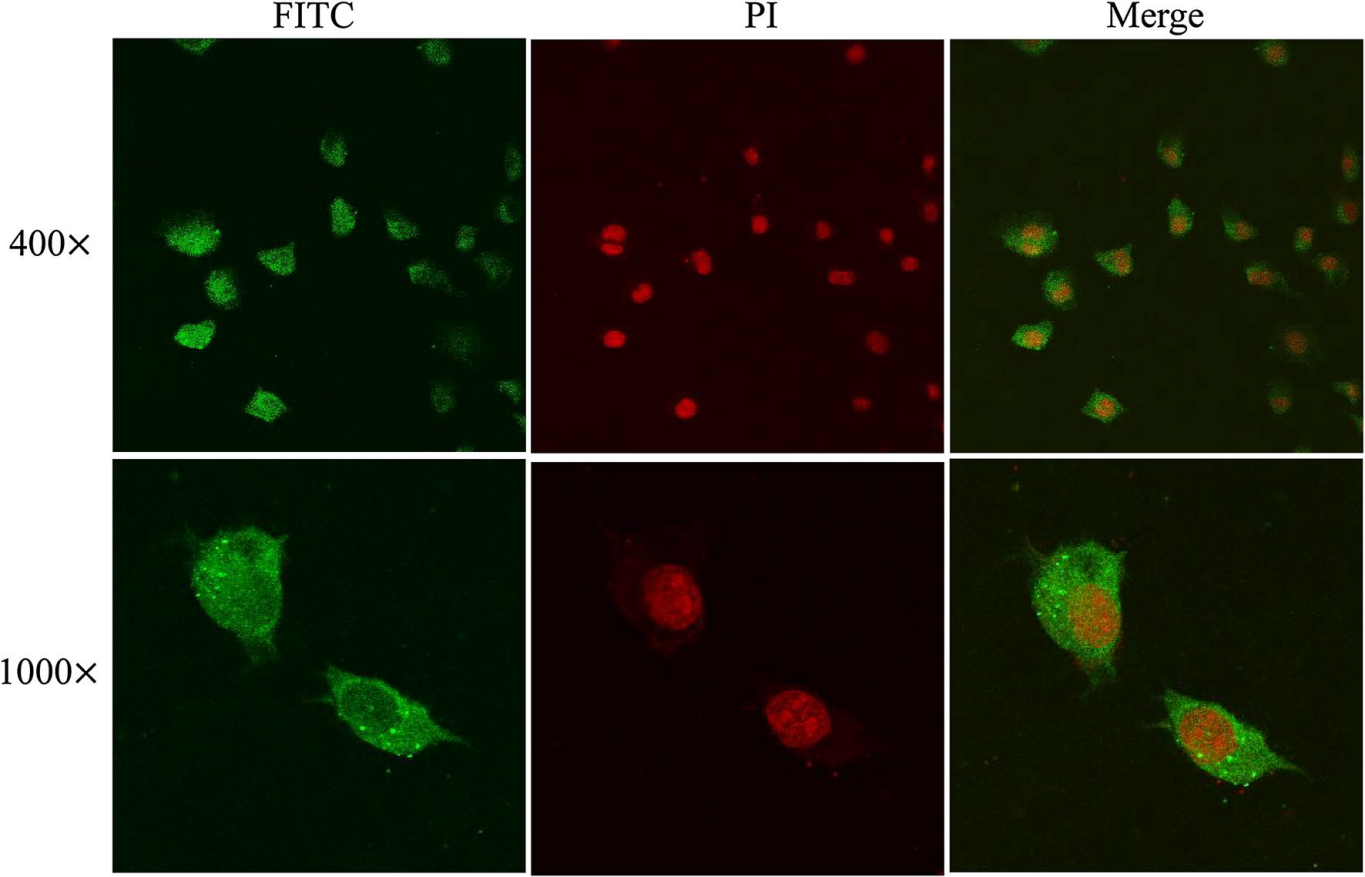
**Subcellular localization of rTsgal binding to IECs by confocal microscopy.** The IECs were pre-incubated with rTsgal for 2 h at 37 °C, then incubated with anti-rTsgal serum, and finally stained with goat anti-mouse IgG-FITC conjugate, and counterstained with propidium iodide (PI).

After the IECs were incubated with rTsgal, the fluorescent staining was detected on the surface of the IECs due to anti-rTsgal serum or infection serum, but not due to normal serum (Figure 8). Furthermore, no staining of C2C12 cells incubated with rTsgal was observed by either anti-Tsgal serum or infection serum. Confocal microscopy revealed that rTsgal could bind to the IECs; the binding sites were located at the membrane and cytoplasm of the IECs (Figure 9).

### Hemagglutination activity of rTsgal and sugar inhibition assay

**Figure 10:**
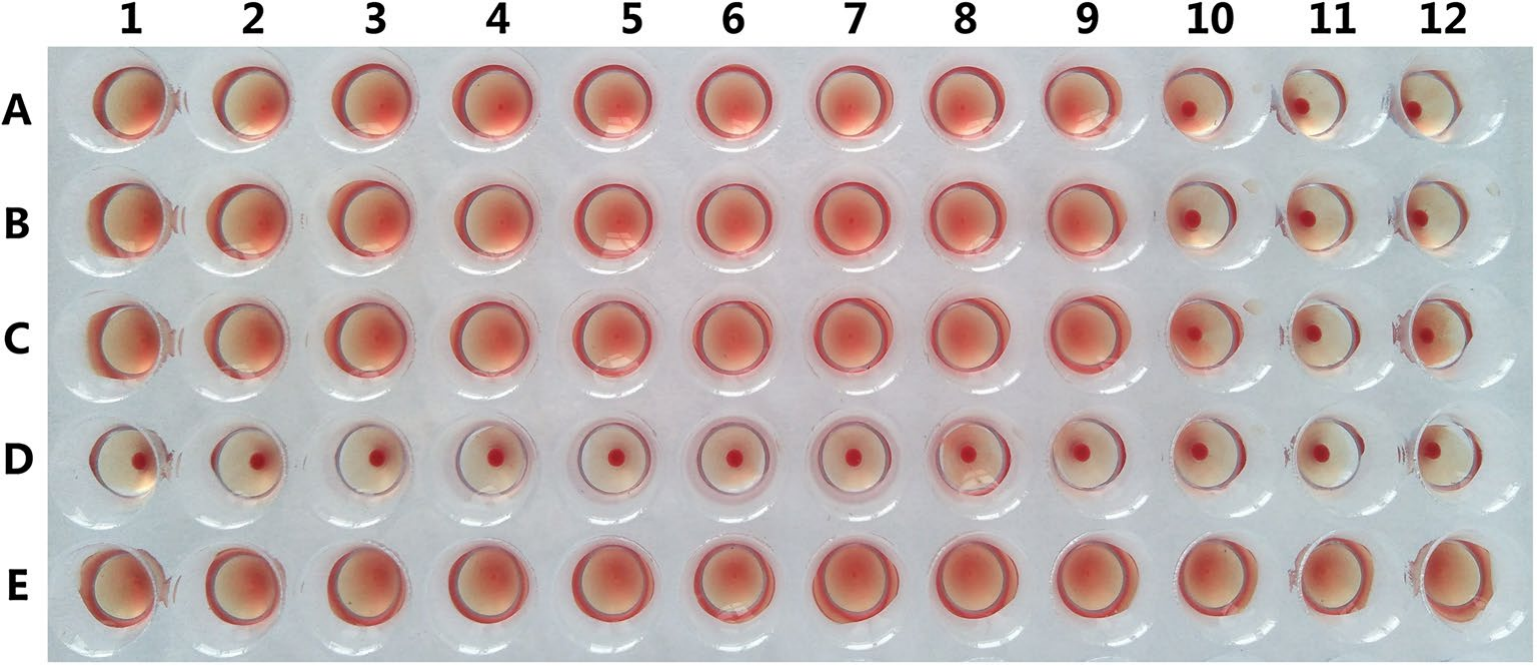
**Inhibition of different carbohydrate on rTsgal hemagglutinating function to human type B erythrocytes.** Lane 1–3: glucose; lane 4–6: sucrose; lane 7–9: maltose; lane 10–12: lactose. A–C Different concentration of various carbohydrates, A = 400 mM, B = 200 mM and C = 100 mM. D: 200 mM carbohydrate, rTsgal was replaced by PBS. E: carbohydrate was replaced by PBS.

The rTsgal was used for the hemagglutination and sugar inhibition assays, the results revealed that rTsgal has the hemagglutinating function, the minimum concentration of rTsgal agglutinating to human type A, B, AB and O erythrocytes was 200, 40, 100 and 100 μg/mL, respectively; the minimum concentration to mouse and rabbit erythrocytes was 100 μg/mL. In the sugar inhibition assay, lactose is the only carbohydrate which can inhibit the agglutination of human type B erythrocytes by rTsgal (Figure 10).

### Promotion or inhibition of rTsgal or anti-rTsgal serum on larval invasion of IECs

**Figure 11:**
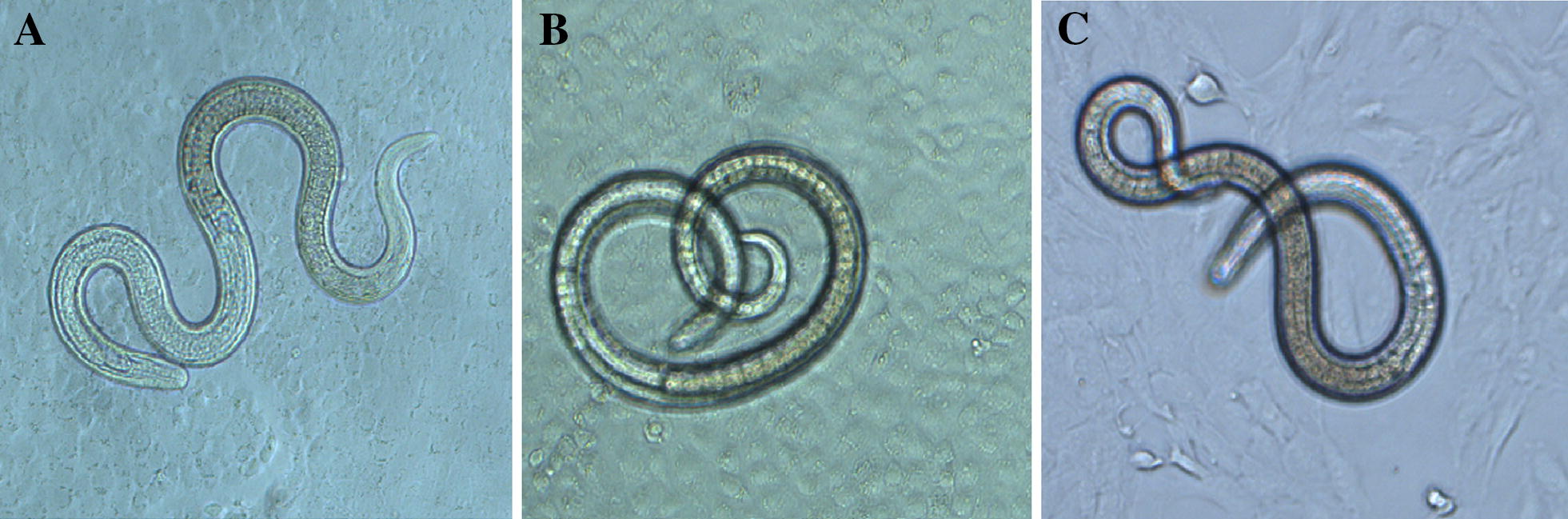
**The invasion process of the IEC monolayer by** ***T. spiralis*** **larvae.** The larvae are inoculated onto the IECs and the invasion process was observed under microscope at 2 h after incubation. **A** Larva invaded the IEC monolayer was mobile and its migrating trail was observed. **B** Non-invaded larva was coiled. **C** Non-invaded larva in C2C12 monolayer.

**Figure 12:**
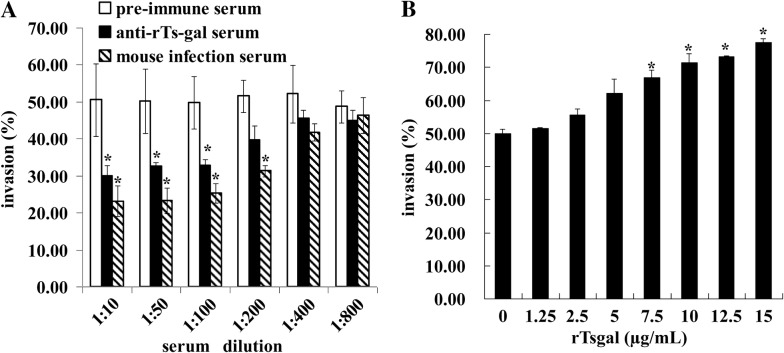
**The inhibition or promotion of** ***T. spiralis*** **larval invasion of IEC monolayer by anti-rTsgal serum or rTsgal.** **A** Inhibition of larval invasion of monolayer by anti-rTsgal serum. One hundred larvae were added into medium and then covered the cell monolayer, different dilutions of anti-rTsgal serum, infection serum or normal serum was used in this experiment and the larval invasion rate was observed 2 h after incubation. **B** The promotion of larvae invasion of IEC monolayer by rTsgal. Different dose of the rTsgal was added into medium, the larval invasion was observed 2 h after cultivation. The larval invasion rate is expressed as the percentages of the larvae penetrated into monolayer and shown as the mean ± SD of three independent experiments. Significant differences (*P *< 0.01) were marked with asterisks (*) relative to normal serum group or control group without rTsgal.

After incubation with the IEC monolayer at 37 °C for 2 h, the IIL invaded the monolayer were observed and counted (Figure 11). While 1:100 dilutions of anti-rTsgal serum, infection serum and pre-immune serum were supplemented into the medium and incubated for 2 h, larvae invaded in monolayer was 32.83, 25.38 and 49.69%, respectively (*χ*^*2*^= 195.915, *P *=0). The inhibition of anti-rTsgal serum (1:10–1:100) on the invasion was evidently higher than those of pre-immune serum (*P *= 0) (Figure 12A). Moreover, when the rTsgal was added into the medium and incubated at 37 °C for 2 h, obvious promotion of rTsgal on larval invasion of the monolayer was found, and this promotion was the rTsgal dose-dependent (*P* = 0), and exhibited an increase trend with the increase of the rTsgal dose (*F *= 45.458, *P *=0) (Figure 12B).

## Discussion

Galectin was widely distributed from vertebrate to invertebrate, even in fungi. In parasites, more and more galectins have been identified and exert a significant impact in the interaction between parasite and host. Helminth infection can also be modulated by the galectins of hosts and vectors, such as *H*. *contortus* [52], *Leishmania major* [53] and *Schistosoma mansoni* [54]. The galectins have also been isolated from nematode parasite *Teladorsagia circumcincta* and *Toxascaris leonina*, their structures and function has been characterized [55–57]. However, the galectins of *T. spiralis* have not been reported up to now.

In the present study, the functional domain of Tsgal gene was cloned from *T. spiralis* ML and expressed successfully by using *E. coli* expression system, the MW of the rTsgal protein is approximately 29.1 kDa in line with the expected size. A 15 amino acids signal peptide was predicted in Tsgal gene, while most galectins lack a typical secretion signal peptide [58]. The Tsgal is identified as TR-galectin because of the two CRD structures. The deduced amino acid sequence of Tsgal had 97.1% identity with the galectin of *T. nativa*, while the Tsgal had 22.1% with galectin-6 of mice and 20.8% identity with galectin-8 of human. It is speculated that the Tsgal from *T. spiralis* cuticles may mimic the galectins of the host as galectin homologues to perform similar function of the mammals.

Western blot results revealed that the native Tsgal protein was identified in somatic proteins of all *T. spiralis* lifecycle stages (ML, IIL, 3 and 6 days AW, and NBL) by anti-Tsgal serum, demonstrating the Tsgal is a somatic protein component of the parasite. RT-PCR results revealed that Tsgal transcription was observed at all *T. spiralis* phases (ML, IIL, 3 and 6 days AW, and NBL). The IFA results revealed that Tsgal expression was detected at all *T. spiralis* various stages, immunostaining was primarily distributed at the surfaces, cuticles, stichosomes and embryos of this nematode. The results suggested that the Tsgal might play some roles as a *Trichinella* surface protein for larval invasion of host’s small intestine [38]. Parasite surface proteins are directly exposed to the host’s immune system and are the pivotal target antigenic molecules. Moreover, the some worm surface proteins may interact with the host to modify the nematode itself and its environment by modulating host’s immune response for immune evasion or even regulating host cell gene expression (such as skeletal muscle myogenesis, development and regeneration), to conduce to the larval invasion, development and survival [59–61].

In the process of larval invasion of the host intestinal mucosa, some proteins secreted from intestinal parasites bond to enterocytes. These proteins may play some functions in the interaction between parasites and host and benefit the parasite survival. Previous studies demonstrated nudix hydrolase secreted by *T. spiralis* (TsNd) bond specifically to the membranes of IECs and facilitated larval invasion and development [29]. Our results of far Western blot confirmed the protein interaction between rTsgal and IECs, ten protein bands of IEC lysates pre-incubated with rTsgal were probed by anti-rTsgal serum. The results of IFA and confocal microscopy indicated that the rTsgal specifically bound to IECs, and the binding sites were located at the membrane and cytoplasm of the IECs. The IEC proteins binding with Tsgal need to be characterized in further experiment. The CRD of *Entamoeba histolytica* Gal/GalNAC lectin was reported to bind to TLR2 and TLR4 in human colonic cells, and activated their signal pathway and then enhanced adhesion of trophozoites to the cells [62]. The galectin from *H. contortus* was identified to have function in immune system by binding with glycoproteins on PBMC, and the glycoproteins may play an important part in cell migration, phagocytosis, and the secretion of the cytokines [63].

The hemagglutination activity of rTsgal was also identified in our study. The rTsgal displayed hemagglutinating activities for four kinds of erythrocytes (A, B, O AB types) of humans as well as those of rabbit and mice. For agglutinating to human type B erythrocytes, the minimum concentration of rTsgal was 40 μg/mL, while the lowest dose of *Toxascaris leonine* galectin is 62.5 μg/mL, and the mouse galectin is 20 μg/mL [20]. Although the carbohydrate binding ability of rTsgal is not very prominent, the carbohydrate-binding activity of rTsgal was exited and further confirmed by sugar inhibiting assay. Out of four carbohydrates, lactose was the only carbohydrate which inhibited the agglutination of human type B erythrocytes by rTsgal. The rTsgal specially binding to lactose was most likely related with their structure [56].

In addition, the results of in vitro invasion assay revealed that rTsgal facilitated the larval invasion of IECs, while anti-rTsgal antibodies inhibited the larval invasion in a dose-dependent mode. The facilitation is likely connected with the tissue structure changes by degrading the ECM of IECs or activating the cell signal pathways, which assisted in parasite invasion of the host’s cells, as the galectins of *D. immitis* and *E. histolytica* [18, 62]. It has been reported that some antibodies against recombinant *Trichinella* proteins protected the intestinal epithelium from larval invasion [34, 49, 64, 65]. The inhibition mechanism of larval invasion of IECs could be owing to the formation immune complex of Tsgal and anti-Tsgal antibody in larval cephalosome, which physically blocked the direct contact between the larvae and host’s cells, and inhibited the larval invasion [66, 67]. The in vivo biological function of the Tsgal need to be further investigated in animal experiment.

In conclusion, the Tsgal was expressed during all *T. spiralis* various phases and located mainly in the surfaces, cuticles and stichosomes of this nematode. The rTsgal had hemagglutinating activities to erythrocytes and bond specifically to IEC membrane and cytoplasm. The rTsgal protein could promote the larval invasion of IEC monolayer, while the anti-rTsgal serum inhibited the larval invasion of the monolayer in dose-dependent mode. These results indicated that the Tsgal might interact with IECs and participate in *T. spiralis* invasion of host’s intestinal epithelium in early infection stage.

## Abbreviations

AW: adult worms
CRD: carbon recognized domain
dpi: days post-infection
DAB: 3, 3′-diaminobenzidine tetrahydrochloride
DSS: dextran sodium sulfate
ECM: extracellular matrix
ES: excretory–secretory
G3PDH: glyceraldehyde 3-phosphate dehydrogenase
hpi: hour post-infection
IBD: inflammatory bowel disease
IEC: intestinal epithelial cell
IFA: immunofluorescent assay
IIL: intestinal infective larvae
IPTG: isopropyl β-d-1-thiogalactopyranoside
ML: muscle larvae
NBL: newborn larvae
OPD: *o*-phenylenediamine dihydrochloride
PI: propidium iodide
PVDF: polyvinylidene difluoride
SD: standard deviation
TBS: tris-buffered saline
Tsgal: *Trichinella spiralis* galectin
TsNd: *T. spiralis* nudix hydrolase
